# Efficacy and safety of hyaluronic-polidocanol foam in sclerotherapy for head and neck venous malformations

**DOI:** 10.3389/fneur.2024.1444896

**Published:** 2024-08-16

**Authors:** Zhaoyang Sun, Yiran Liu, Anwei Chen, Tao Wang, Shaohua Liu

**Affiliations:** ^1^Department of Oral and Maxillofacial Surgery, Qilu Hospital of Shandong University, Jinan, Shandong, China; ^2^Department of Oral and Maxillofacial Surgery, Weifang People’s Hospital, Weifang, Shandong, China; ^3^Department of Endocrinology, Qilu Hospital of Shandong University, Cheeloo College of Medicine, Shandong University, Jinan, Shandong, China

**Keywords:** polidocanol foam, sclerotherapy, hyaluronic acid, venous malformations, sclerosant

## Abstract

**Background:**

Foam sclerotherapy is currently the first-line treatment for venous malformations (VMs). Hyaluronic acid-polidocanol (HA-POL) foam has been used in the treatment of head and neck VMs recently; however, its clinical efficacy and safety have yet to be further evaluated, and the impact of age and other related factors on its safety is still unclear.

**Objective:**

To assess the efficacy and safety of HA-POL foam in the treatment of head and neck VMs.

**Methods and materials:**

We performed a single-center retrospective review of all patients with VMs involving the head and neck region undergoing HA-POL foam sclerotherapy from February 2015 to February 2022 in the Oral and Maxillofacial Surgery Department of Qilu Hospital Shandong University. Patients’ medical records were collected and all patients enrolled were followed up for 1–6 months (group 1), part of them were followed up for 3–9 years (group 2).

**Results:**

A total of 223 patients with head and neck VMs were enrolled in the study, with 36 patients who were followed for 3–9 years. Total response rate in group 1 was 96.41% (*n* = 215), of which 30.94% (*n* = 69) of the patients met the criteria of “resolution,” and 65.47% (*n* = 146) of the patients had “significant improvement.” In group 2, the total response rate was 72.22% (*n* = 26), of which the rates of the patients met the criteria of “resolution” and patients had “significant improvement” were all 36.11% (*n* = 13)0.144 (64.57%) patients experienced complications like localized swelling, pain and fever, and no serious complications occurred. The risk of developing complications after treatment was independent of age, and was weakly associated with the dose of HA-POL foam.

**Conclusion:**

The HA-POL foam sclerotherapy is safe and effective in the treatment of head and neck VMs.

## 1 Introduction

The treatment of head and neck venous malformations (VMs) requires multidisciplinary collaboration, and available treatments include surgery, laser therapy, sclerotherapy, and vascular intervention (1, 2). Extensive or radical surgical resection is often difficult to achieve due to the complicated histological structures involved in the head and neck region (3).

Foam sclerotherapy is currently the preferred therapy for VMs (4), which is characterized by minimal invasive operation, high efficiency and safety. Polidocanol is a widely used foam sclerosant agent.

It has been recognized that the sclerosing ability is highly influenced by foam stability (5, 6), the addition of Hyaluronic acid (HA) to polidocanol foam (HA-POL foam) can effectively prolong the stability of polidocanol foam (7–9). However, the safety and efficacy of HA-POL foam has been less studied. A single-center retrospective study including 70 patients conducted by Chen et al. confirmed that the application of HA-POL foam sclerotherapy for the treatment of head and neck VMs is safe and effective (10). However, the sample size of this study was small, resulting in a relatively low level of evidence.

Considering that serious adverse events of foam sclerotherapy (11), such as deep vein thrombosis, superficial vein thrombosis, and pulmonary embolism, already have a high morbidity in the elderly (12), the safety of HA-POL foam sclerotherapy in the elderly should be given special attention. Similarly, the characteristics of children in terms of physiology, pharmacology, growth and development differ greatly from those of adults, and guiding the use of drugs in children on basis of the results of safety and efficacy studies based on adults may lead to unpredictable and serious consequences (13, 14). Therefore, in view of the particularity of children and the elderly, it is necessary to explore the relationship between age and the efficacy and safety in foam sclerotherapy.

Therefore, we performed a retrospective review with a larger sample size, to assess the efficacy and safety of HA-POL foam in the treatment of head and neck VMs, especially in the children and the aged.

## 2 Methods and materials

### 2.1 Study design

A single-center retrospective study was conducted on patients with head and neck VMs treated with HA-POL foam sclerotherapy at the Department of Oral and Maxillofacial Surgery, Qilu Hospital, Shandong University, between February 2015 and February 2022. The study was approved by the Ethics Committee of Qilu Hospital, Shandong University. In group 1, all enrolled patients were classified into 3 age groups, 0–18 years, 19–59 years and ≥ 60 years according to the age of the first visit. The diagnosis of VMs was based on medical history, clinical manifestations, MRI, ultrasound, and other ancillary examination findings according to the classification criteria of the International Society for the Study of Vascular Anomalies (ISSVA) on vascular diseases. Besides, the following patients were excluded: (1) Patients with other vascular diseases or VMs in other sites treated at the same time; (2) Patients treated with other sclerosants or other forms of treatment within 6 months (including 6 months).

### 2.2 Foam preparation

The Tessari method was adopted for the preparation of HA-POL foam: two 10 mL sterile disposable syringes (WEGO, Weihai, China) were connected at 90° through a medical three-way valve (B. Braun Melsungen AG 34209 Melsungen, Germany). 2 mL of 1% POL liquid (SHANXI TIANYU Pharmaceutical Co., Ltd) and 0.1 mL of 1% HA (20 mg/2 mL Sofast, Sodium Hyaluronate Injection, Shandong Bausch-fruida Pharmaceutical Co Ltd., Shandong, China) were drawn into one of the 10 mL syringes, and the other was filled with 8 mL of air. The two syringes were pushed back and forth for at least 20 times at room temperature in a 4:1 air-liquid ratio to produce HA-POL foam, and the foam was used immediately for sclerotherapy.

### 2.3 Sclerotherapy process

Sclerotherapy is performed using either a two-needle technique or a multiple-needle technique using winged needles (Shandong Ande Healthcare Apparatus Co., Ltd., China), depending on the patients age and the size of the lesion. The treatment was aided with ultrasound-guiding if necessary. One sterile winged needle was inserted into the lesion, with venous blood was withdrawn to make sure the correct position. A second winged needle connected with a 10 mL syringe would be punctured into the other part of the lesion before the HA-POL foam was slowly injected into the lesion, the injection would cease until foam was observed in the second syringe. If the treatment was ultrasound-guided, the injection was stopped when the lesion was full filled with HA-POL foam under ultrasound, and the dose of foam sclerosing agent used was recorded. The dose of foam for a single sclerotherapy injection should not exceed 30 mL. After injection, sterile gauze was used to compress the entry point by mild pressure to avoid spillage of sclerosing agent through the entry point and to control bleeding. The patient was asked to rest for at least 10 min after treatment. The interval between treatments is 1 month. Signs of discontinuation of sclerotherapy include: (1) clinical assessment that the lesion has disappeared or decreased in size, the symptoms have disappeared or resolved, and that continuation of the treatment will not result in a better therapeutic outcome; and (2) the patient or the patients’ guardian voluntarily requesting. All patients were followed up for 1–6 months (group 1), part of them were followed for 3–9 years (group 2).

### 2.4 Clinical efficacy and safety assessment

We obtained the following data from the patients’ medical records, and follow-up results: gender, age at first treatment, location of the lesions, course of treatments, volume of foam sclerosant used, treatment response, and complications of post-treatment.

After sclerotherapy, the efficacy and safety were evaluated at the last follow-up visit. Clinical efficacy was evaluated according to a modified scoring system proposed by Achauer et al.: “Cure” indicated that the clinical manifestations of VMs had completely disappeared; “Significant improvement” indicates a continuous reduction in lesion size by 50% or more, or that the original dysfunction had completely returned to normal; “no effect” indicates a recurrence or no improvement in the size and the functional impairment caused by the lesion.

The occurrence of complications during and after sclerotherapy were collected in the medical records and during follow-up, and the types, time of occurrence of complications were recorded for safety assessment.

### 2.5 Statistical analysis

Descriptive statistics were used in the statistical analysis, where continuous variables were described by means ± standard deviation and categorical variables were described by numerical values (percentages). We applied SPSS 23.0 software package to analyze the data by statistical methods of binary Logistics linear regression and Pearson correlation analysis.

## 3 Results

### 3.1 Patients’ characteristics

Total of 223 patients were enrolled in group 1 (Table 1), of whom 78 patients were male and 145 patients were female. The average age of the patients at first treatment was 29.91. Number of patients in age group of 0–18 years, 19–59 years and ≥ 60 years were 75,137 and 11 patients, respectively. There were total 36 patients underwent further follow-up in group 2, details are given in Table 2.

**Table 1:** Clinical date of patients with head and neck venous malformations.

Characteristic	Number	%	Standard deviation
Sex (*n* = 223)			
Female	145	65.02%	
Male	78	34.98%	
Age (*n* = 223)	29.91 (0–75)		17.77
Age group (*n* = 223)			
0–18 years	75	33.60%	
19–59 years	137	61.43%	
≥60 years	11	4.93%	
Treatment session (*n* = 380)	1.70		1.41
Volume of foam (*n* = 380)	9.82		6.31

**Table 2:** Clinical date of patients with head and neck venous malformations (group 2).

Characteristic	Number	%
Sex (*n* = 36)		
Female	27	75.00%
Male	9	25.00%
Age (*n* = 36)	30.25	
Treatment session (*n* = 51)	1.42	
Volume of foam	10.42	
Cure	13	36.11%
Significant improvement	13	36.11%
No effect	10	27.78%

Sites of lesions included the face, tongue, lips, neck, parotid gland, periorbital, nose, mental region, palate, temporal region, floor of the mouth, gums, and oropharynx, with 32 of the patients’ lesions were multiple (Table 3).

**Table 3:** Locations of the head and neck venous malformation lesions.

Location of VMs	Number	%
Face	73	32.74%
Tongue	39	17.49%
Lips	41	18.39%
Multiple	32	14.35%
Neck	8	3.59%
Parotid region	5	2.24%
Periorbital	5	2.24%
Nose	4	1.79%
Mental region	4	1.79%
Temporal region	3	1.35%
Palate	3	1.35%
Floor of mouth	3	1.35%
Gums	2	0.90%
Oropharynx	1	0.90%

In group 1, there was a total of 380 treatment sessions for all 223 patients (Table 1), with an average of 1.7 sessions each (range 1–12). The average volume of HA-POL foam used per treatment was 9.82 mL, ranging from 1.20 mL to 30.00 mL.

### 3.2 Clinical efficacy

The overall efficiency in group 1 was 96.41% (*n* = 215), of which 30.94% (*n* = 69) of the patients achieved “Cure,” and 65.47% (*n* = 146) achieved “Significant improvement.” There were 3.59% (*n* = 8) patients had no response. Results of Pearson correlation analysis showed that age was not related to the efficacy of treatment [point two-column correlation coefficient: *R* = 0.078, *p* > 0.05].

Of the 8 patients who were “no effect,” 3 patients had multiple lesions, accounting for 37.50%. Therefore, it was considered that the multiple lesions might affect the results of the treatment. Therefore, the patients were categorized into “Effective(including “Cure” and “Significant improved)” (*n* = 215) and “Ineffective” (*n* = 8) groups according to the treatment effect, the statistical results showed that whether the lesions were multiple or not did not affect the efficacy of treatment, and the difference was not statistically significant [9.38% vs. 2.62%, OR = 3.848, *95%CI* (0.873, 16.971), *p*>0.05], and the lesion sites did not affect the efficacy(*p*>0.05).

In group 2, the overall efficiency was 72.22% (*n* = 26), of which the rates of the patients met the criteria of “resolution” and patients had “significant improvement” were all 36.11% (*n* = 13). There were 10 (27.78%) patients who had “no effect,” of which 7 patients had recurrence (Table 2).

### 3.3 Risk assessment of complications

There were 141 patients experienced localized swelling, which was the most common complication. Two patients had localized pain, and 1 patient had fever. All patients experienced gradual relief and recovery within 2–3 days. No patient developed serious complications such as localized skin and mucosal ulcers, pulmonary embolism, allergic and allergic-like reactions, deep vein thrombosis, shock. No patient whose lesion involved parotid area and periorbital area developed complications of facial nerve dysfunction and visual impairment after treatment. Data of complications is detailed in Table 4.

**Table 4:** Complications of HA-POL foam sclerotherapy.

Complication	Number (%)
Localized swelling	141 (63.36%)
Pain	2 (0.90%)
Fever	1 (0.45%)
Skin and mucosal ulcer	0 (0.00%)
Bacterial infection	0 (0.00%)
Cutaneous necrosis	0 (0.00%)
Pulmonary embolism	0 (0.00%)
Anaphylaxis and anaphylactoid reactions	0 (0.00%)
Deep vein thrombosis	0 (0.00%)
Shock	0 (0.00%)
Never damage	0 (0.00%)
Visual impairment	0 (0.00%)

A 27-year-old female patient with lesions in the buccal mucosa who had suffered from shock during pingyangmycin sclerotherapy years ago did not experience any complications. A 35-year-old male patient with a history of uremia received twice treatments, and no complication occurred. The results of the above study demonstrated that the use of HA-POL foam sclerotherapy for the treatment of VMs in the head and neck region is safe.

### 3.4 Correlation analysis between age and safety

We categorized all the patients into three age groups according to their age, 0–18 years, 19–59 years and ≥ 60 years, and compared the post-treatment complications among patients of different age groups, respectively, to investigate the safety of HA-POL foam sclerotherapy. The difference of the risk of complication between three groups was not statistically significant. Results are detailed in Table 5.

**Table 5:** Analysis of relationship between age group and complications after HA-POL foam sclerotherapy.

Age group	Complication (*n* = 141)	No complications (*n* = 82)	Total (*n* = 223)	*p*-value
Age group				0.424
0–18 years	43 (57.33%)	32 (42.67%)	75	0.190
19–59 years	91 (66.42%)	46 (33.58%)	137	
≥60 years	7 (63.64%)	4 (36.34%)	11	0.851

Although patients in the age group of 19–59 years had the highest probability of complications after treatment, followed by the patient of age group ≥60 years, and the patient of age group 0–18 years had the lowest risk, the difference was not statistically significant, and the results of the correlation analysis showed that the risk of complications did not correlate with the patients age [point two-column correlation coefficient: *R* = 0.149, *p* > 0.05]. It is evident that HA-POL foam sclerotherapy for head and neck VMs is safe for all age groups.

### 3.5 Correlation between the volume of HA-POL foam and safety

The mean volume of HA-POL foam used per patient was 9.82 ± 6.31 mL. The single-treatment dose of foam sclerosant used was weakly and positively correlated with the risk of complications [point two-column correlation coefficient: *R* = 0.209, *p* < 0.05]. That is, the higher the volume of HA-POL foam used in a single treatment, the higher the risk of complications such as local swelling and pain after treatment. Therefore, in the clinical application of foam sclerotherapy for the treatment of VMs in the head and neck region, controlling the volume of foam sclerotherapy used in a single treatment helps to improve the safety of the treatment.

## 4 Discussion

The site and size of VMs lesions limits the choice of treatment method, and in this study, the highest percentage of patients with lesions located in the cheeks was 32.74%, followed by the tongue (17.49%) and lips (18.39%). VMs in the head and neck region often involve important anatomical structures, making the lesion cannot be completely resected, and surgery is also accompanied by risks of adjacent nerve damage, bacterial infection and scar formation (2). Percutaneous sclerotherapy is currently the most widely used treatment for VMs (15). In this study, the overall treatment efficiency of HA-POL foam sclerotherapy was 96.41% in group 1, among them, 30.94% of the patients were “cured,” 65.47% were “significantly improved.” We found that whether the lesions involved multiple sites or not did not affect the efficacy of the treatment, and the treatment response of lesions in different site had no difference. The results certified that HA-POL foam sclerotherapy is very effective in the treatment of head and neck VMs, and the therapeutic efficacy is not limited by the complex anatomical structure.

Considering the short follow-up time, we performed a further follow-up study, and finally 36 patients were followed up for 3–9 years. In this group with a long follow-up time, the treatment efficiency decreased significantly, with an overall response rate of 72.22%. It is worth noting that the rate of patients meeting “cure” criteria has increased. Considering fewer patients were enrolled, whether this difference is meaningful needs further investigation. In addition, among the 10 patients had “no effect,” half of the patients reached the “cured” and “significantly improved” criteria after treatment but had recurrence. The high recurrence rate suggests that the reduction in treatment response rate may be closely associated with recurrence, but this also needs to be confirmed by further studies.

Compared with liquid sclerosant, foam sclerosant can achieve better treatment response by increasing the contact area and contact time between the drug and the lesions, and enhance safety by reducing the dose of sclerosant (8, 16). It is believed that increasing the stability of the sclerosing agent foam correlates with better therapeutic outcomes. There are many factors affecting the stability of sclerosant foam (17, 18), and our previous study confirmed that the addition of a small dose of HA can significantly increase the stability of POL foam (9). HA is a linear glycosaminoglycan, as a natural polymer involved in the formation of the extracellular matrix, it is widely found in human body (19, 20). Research has proven that intravenous application of HA is safe for humans (9, 21, 22).

In a study by Chen et al. (10), 70 patients with VMs of the head and neck treated with HA-POL foam sclerotherapy were enrolled and the results of the study showed a 100% total response rate, of which “Resolution” was achieved in 21 cases (30%) and a significant response in 49 patients (70%). Localized swelling was the most common post-treatment complication but it resolved within 2–3 days, and one patient developed localized mucosal ulceration after treatment, with no other serious complications. However, this study included a small sample size and did not mention the efficacy and safety of HA-POL foam sclerotherapy in different age groups, especially in children and the elderly. VMs, as congenital lesions, often occur in children, who have complex physiological, developmental, psychological, and pharmacological characteristics, and their metabolism of specific drugs is different from that of adults, which may lead to poor therapeutic efficacy, side effects, and drug poisoning. S.P. Mooijaart et al. suggested that the physiological differences between the elderly and the young, such as altered renal function, hepatic function, and body composition of the elderly affects the metabolism of the body and the clearance of the drug, in addition to the fact that the elderly patients are exposed to drug interactions due to the simultaneous application of multiple medications (23). Children and the elderly deserve special attention when assessing the safety and efficacy of a drug or treatment. It is necessary to explore the relationship between age and the safety of HA-POL foam sclerotherapy.

In our study, after HA-POL foam sclerotherapy, intra-and post-treatment adverse events occurred in 144 patients (64.57%), of which localized swelling had the highest incidence of 63.36% and was the most common complication after HA-POL foam sclerotherapy, followed by pain and fever, but with a very low percentage of 0.90 and 0.45%, respectively. There were no serious adverse events such as localized skin mucosal ulcers, superficial tissue necrosis, infection, deep vein thrombosis, pulmonary embolism, or shock in this study. All patients presented with uncomfortable symptoms gradually relieved and recovered within 2–3 days. We also categorized the enrolled patients into three age groups: 0–18 years, 19–59 years, and ≥ 60 years, and compared the risk of complication between different groups, respectively. The results showed that the risk of complications after HA-POL foam sclerotherapy was consistent between patients in the 0–18 years age group and the 19–59 years age group, and the difference was not statistically significant. The risk of complications in group of ≥60 years was 63.64%, and all of them were localized swelling and resolved soon after treatment, no serious adverse complications occurred. There was no statistically significant difference in the risk of complications in patients ≥60 years of age compared to patients 19–59 years of age, indicating HA-POL foam sclerotherapy is safe for children and the elderly population as well.

Based on the above findings about different age groups, we further explored the correlation between age and the risk of complications after HA-POL sclerotherapy, and the results showed that there was no correlation between age and the risk of complications, demonstrating the application of HA-POL foam to treat head and neck VMs has a high level of safety for all age groups.

In this study the effect of the dose of foam sclerotherapy used in a single treatment on the safety of the treatment was investigated. The volume of sclerosing agent foam used per treatment was determined by the treating physician based on the size of the lesion, but the maximum volume of foam used in a single session was not to exceed 30 mL to ensure the safety of the treatment. In the 223 patients enrolled in this study, the average HA-POL foam volume used in a single session for each patient was 9.82 mL (from 1.20 mL to 30.00 mL). The results showed that there is a weak positive correlation between the volume of foam sclerosant used in a single treatment and the risk of complications. That means the higher the volume of HA-POL foam sclerosant used in a single treatment, the higher the risk of complications. Therefore, controlling the volume of HA-POL foam in a single treatment is one of the keys to ensure the safety of treatment.

The limitations of this study included the use of a single-center clinical retrospective study, the absence of some case data, and the fact that most of the patients did not undergo a second MRI and ultrasound check after treatment to accurately assess the change in lesion volume. This study did not include patients treated with POL foam sclerotherapy alone to form a controlled trial. Otherwise, the short follow-up time limits the accuracy of the study findings. Although we did further long-term study, the small number of patients enrolled is still insufficient to illustrate treatment efficacy. There is a need for larger prospective clinical studies or randomized controlled trials to provide more convincing results on the efficacy and safety of HA-POL in the treatment of head and neck venous malformations.

## 5 Conclusion

In this study, we demonstrated that the application of HA-POL foam sclerotherapy for the treatment of head and neck VMs is safe and effective, and that the efficacy and safety of this treatment are not affected by age, including children and the elderly. The volume of HA-POL foam used in a single treatment session was weakly correlated with the risk of complications, so careful control of drug dosage can help to improve the safety of the treatment.

## Data Availability

The raw data supporting the conclusions of this article will be made available by the authors, without undue reservation.
